# Rheumatoid arthritis disease activity significantly impacts on the severity of interstitial lung disease

**DOI:** 10.1186/s13075-024-03333-6

**Published:** 2024-05-04

**Authors:** Yuhei Ito, Yasutaka Ichikawa, Shuichi Murashima, Hajime Sakuma, Ayako Nakajima

**Affiliations:** 1 Centre for Rheumatic Diseases, Mie University Hospital, 2-174 Edobashi, Tsu, 514-8507 Mie Japan; 2 Department of Radiology, Mie University Hospital, 2-174 Edobashi, Tsu, 514-8507 Japan

**Keywords:** Connective tissue disease, Disease activity, Interstitial lung disease, Rheumatoid arthritis

## Abstract

**Objectives:**

Rheumatoid arthritis (RA) related interstitial lung disease (ILD) impacts on the treatment strategy and its prognosis in patients with RA. However, the relationship between RA disease activity and the severity of comorbid ILD has not been fully investigated. This study aimed to investigate the impact of RA disease activity on the severity of comorbid ILD in detail based on currently established visual scoring method along with physiological severity.

**Methods:**

Consecutive patients with RA visiting to our Rheumatology Centre between December 2020 and December 2023 were analysed. The radiological severity of ILD was evaluated by averaging the extent of the combined lesion of ground glass opacity, reticulation and honeycombing in 5% increments in six representative high-resolution computed tomography slices ranging from 0% (no involvement) to 100% (all lung fields affected) according to Goh and Walsh’s method. Associations between the radiological and physiological severity of ILD and patients’ features were investigated using linear regression analysis.

**Results:**

Among 124 patients (32 men, 92 women), the median age was 70 years, and the median disease duration was 2.92 years. Radiological severity of ILD was 0% (without ILD) in 107 (86.2%), ILD with extent < 10% in nine (7.2%), ILD with extent ≥10% and < 20% in three (2.4%), ILD with extent ≥20% in five (4.0%). Both disease activity score (DAS)28-erythrocyte sedimentation rate (ESR) (standardized coefficient = 0.199, *P* = 0.03) and rheumatoid factor titre (standardized coefficient = 0.247, *P* = 0.01) were significantly associated with the radiological quantitative severity of ILD in multivariate analysis adjusted for age, sex, disease duration, smoking status and anti-citrullinated peptide antibody titre. DAS28-ESR was significantly associated with forced vital capacity% predicted (standardized coefficient = -0.230, *P* = 0.047).

**Conclusions:**

Disease activity of RA was significantly associated with the severity of RA-ILD both radiologically and physiologically.

**Supplementary Information:**

The online version contains supplementary material available at 10.1186/s13075-024-03333-6.

## Introduction

Interstitial lung disease (ILD) is an extra-articular comorbidity of rheumatoid arthritis (RA) which negatively impacts the prognosis of patients with RA [1, 2]. Currently, clinical evaluation for RA-ILD severity is based on the combination of pulmonary function tests and radiological extent of ILD on high-resolution computed tomography (HRCT) [1]. Impaired pulmonary function tests are associated with poor survival of patients with RA-ILD and are widely used to clinically assess the prognosis of RA-ILD [1, 3]. Several studies have reported on the usefulness of radiological extent of ILD as a prognostic predictor in patients with RA-ILD [4–8]. In addition, it has become important to consider the therapeutic options for RA based on the radiological severity of RA-ILD [9]. The relationship between RA disease activity and the presence or development of ILD has been reported in several studies; however, these studies focused only on the presence or absence of ILD and not on the quantitative severity of ILD [10–12]. To date, one paper has assessed the disease activity of RA and the severity of RA-ILD [13]. However, this paper is limited in that the evaluation for the extent of ILD was focusing only on ground glass opacity (GGO) but not on reticulation or honeycombing, in 3 selected slices which is rough compared to the currently established Goh and Walsh’s scoring method [14, 15] that evaluates 5–6 slices. Furthermore, the physiological severity of RA-ILD was not investigated.

Our study aimed to investigate the association between RA disease activity and ILD severity by focusing primarily on the radiological quantitative severity of ILD, based on the currently established visual scoring method in which combined extent of GGO, reticulation and honeycombing are evaluated in six slices [6, 14, 15], in addition to physiological severity.

## Patients and methods

### Data collection

A database of patients with connective tissue disease (CTD) was created by enrolling consecutive patients who visited our Rheumatology Centre between December 2020 and March 2023 to analyse CTD-ILD. This study conformed to the Declaration of Helsinki guidelines and was approved by the institutional review board of the Mie University Hospital (Approval number: H2020-242). Only patients who provided informed consent for participation were enrolled in the study.

Subsequently, patients who met the 2010 Rheumatoid Arthritis Classification criteria [16] were selected from this database. The baseline clinico-demographic data of all patients were collected from their medical charts. Data included the following patient characteristics: age, sex, disease duration, smoking status, modified Medical Research Council dyspnoea scale, presence of cough, swollen and tender joint counts, Steinbrocker stage based on radiographs, laboratory data (erythrocyte sedimentation rate (ESR), C-reactive protein, rheumatoid factor (RF), antinuclear antibody (ANA), anti-cyclic citrullinated peptide (CCP) antibody, Krebs von den Lungen-6 antigen (KL-6), and surfactant-associated protein D). All patients were evaluated for disease activity score (DAS) 28–ESR and clinical disease activity index (CDAI) at registration. All patients basically underwent HRCT at registration. If HRCT was performed within 6 months before registration, that HRCT was used for analysis. Patients with ILD underwent pulmonary function tests (forced vital capacity (FVC) and FVC% predicted) if they consented. Pulmonary function tests were not mandatory for patients without ILD. Acute exacerbation of ILD was assessed using pre-established criteria [17]. Patients with concomitant CTDs including systemic sclerosis [18], systemic lupus erythematosus [19], polymyositis /dermatomyositis [20] and mixed connective tissue disease [21, 22] were excluded from this analysis.

### HRCT protocol and image evaluation

#### Image evaluation

The extent of ILD was estimated based on Goh et al. and Walsh et al.’s methods [14, 15]. A detailed description of the HRCT protocol, image evaluation, and methods for calculating the disease extent are provided in Supplementary Data S1. Briefly, the extent of disease was calculated by predetermined six representative slices, calculating the percentage of interstitial abnormalities in each slice, and dividing the total score by six [14]. The quantitative extent of ILD was calculated by assessing areas wherein any of the following three interstitial abnormalities were present: GGO, reticulation, and honeycombing [15]. The extent of ILD was expressed ranging from 0% (no involvement) to 100% (all lung fields affected). An expert certified pulmonologist and rheumatologist (Y.I) and an expert radiologist specialized in chest CT (S.M) with 13 and 36 years of experience, respectively, scored the HRCT images. The mean of both readers’ scores was used for the analysis.

#### Presence of ILD

In this study, patients with ILD were defined as those with probable or definite ILD according to Bongartz et al.’s criteria, with minor modifications [23] (Supplementary Table S1). For patients without ILD, ILD scores were calculated as zero. Patients with ILD were further stratified according to its extent of < 10%, ≥10% and < 20% and ≥20% based on previous reports [6, 15, 24].

#### Statistical analyses

Baseline characteristics were presented as median (interquartile range (IQR)) and number (percentage) for continuous and categorical variables, respectively. Associations between the quantitative extent of ILD and the following covariates were assessed using univariate linear regression analysis: male sex, age, disease duration, smoking status, DAS28-ESR, RF and anti-CCP antibody titre at registration. If the titres of RF and anti-CCP antibodies were below or above a certain value, e.g., < 5 and > 500, they were calculated as 5 and 500, respectively. Associations between the radiological quantitative extent of ILD and DAS28-ESR were assessed using multivariate linear regression analysis adjusted for age, male sex, disease duration, smoking status, RF and anti-CCP antibody titre. Similarly, associations between the value of FVC% predicted and the same covariates were assessed using univariate and multivariate linear regression analyses. Spearman’s correlation analysis was used to evaluate inter-reader agreement of ILD extent, which was classified as ‘slight’ (κ = 0.00–0.20), ‘fair’ (κ = 0.21–0.40), ‘moderate’ (κ = 0.41– 0.60), ‘substantial’ (κ = 0.61–0.80), or ‘nearly perfect’ (κ = 0.81–1.00). *P* < 0.05 was considered statistically significant. All statistical analyses were performed using R (The R Foundation for Statistical Computing V.4.2.2, Vienna, Austria).

## Results

### Baseline characteristics of study participants

Participants’ baseline characteristics are listed in Table 1. Finally, 124 patients (32 men, 92 women) were included. The median age was 70.0 (IQR 59.0, 75.0) years, and the median disease duration was 2.92 (IQR 1.40, 8.75) years. Seventy-five (60.9%) were RF positive and 62 (50.4%) were anti-CCP antibody positive, and 36 (29.0%) were ANA positive. The median RF titre was 28 U/mL (IQR 5, 139), and the median anti-CCP antibody titre was 4.6 U/mL (IQR 0.5, 165.9). The median values of DAS28-ESR were 2.34 (IQR 1.54, 3.36). Pulmonary function tests were available for 83 participants, and the median value of FVC% predicted was 100.8% (IQR 91.2%, 112.0%). According to HRCT assessment, 17 was classified as having ILD and 16 (12.9%) showed RA-associated bronchial disease (bronchiolitis or bronchiectasis). Four patients (3.20%) were diagnosed as having chronic obstructive pulmonary disease at registration, and none of these patients were classified as having ILD. At registration, the proportion of patients receiving concomitant medications was as follows: glucocorticoids 45.1%, methotrexate 62.9%, tumor necrosis factor inhibitors 8.8%, interleukin-6 inhibitors 20.1%, abatacept 4.8% and Janus kinase (JAK) inhibitors 12.1%.

**Table 1:** Characteristics of the patients

	Total (*n* = 124)
Demographics	
Age (years), median (IQR)	70.0 (59.0, 75.0)
Male, n (%)	32 (25.8%)
Disease duration (years), median (IQR)	2.92 (1.40, 8.75)
Ever smoker, n (%)	46 (37.1%)
mMRC, 0 / 1 / 2 / 3 / 4, n	90 / 23 / 4 / 6 / 1
Cough, present, n (%)	25 (18.6%)
Steinbrocker stage I / II / III / IV	91 / 9 / 8 / 15
Steinbrocker stage ≥II	32 (25.8%)
Laboratory data	
WBC (/µL), median (IQR)	5640.0 (4438.0, 7575.0)
Lymphocyte (/µL), median (IQR)	1420.0 (1017.0, 1797.0)
Haemoglobin (g/dL), median (IQR)	13.2 (12.4, 14.2)
LDH (U/L), median (IQR)	202.5 (178.5, 238.5)
ALP (U/L), median (IQR)	74.0 (61.0, 92.0)
KL-6 (IU/mL), median (IQR)	230.0 (180.8, 326.8)
SP-D (ng/mL), median (IQR)	67.1 (45.3, 98.9)
ANA positive (≥1: 80), n (%)	36 (29.0%)
Rheumatoid factor, positive, n (%)	75 (60.9%)
Rheumatoid factor, titre (U/mL)	28 [5, 139]
Anti-CCP antibody, positive, n (%)	62 (50.4%)
Anti-CCP antibody, titre (U/mL)	4.6 [0.5, 165.9]
DAS28-ESR, median (IQR)	2.34 (1.54, 3.36)
Pulmonary function tests, n	*n* = 83
Forced vital capacity (L), median (IQR)	2.65 (2.22, 3.07)
Forced vital capacity percent predicted (%), median (IQR)	100.8 (91.2, 112.0)
HRCT assessment	
ILD present, n (%)	17 (13.7%)
Treatment	
Glucocorticoids, n (%)	56 (45.1%)
Methotrexate, n (%)	78 (62.9%)
bDMARDs, n (%)	43 (34.6%)
TNF inhibitors, n (%)	11 (8.8%)
IL-6 inhibitors, n (%)	25 (20.1%)
Abatacept, n (%)	6 (4.8%)
JAK inhibitors, n (%)	15 (12.1%)

Abbreviations: ALP, alkaline phosphatase; ANA, antinuclear antibody; Anti-CCP antibody, anti-cyclic citrullinated peptide antibody; bDMARDs, biological disease-modifying anti-rheumatic drugs; CDAI, clinical disease activity index; CTD, connective tissue disease; DAS28-ESR, disease activity score 28-erythrocyte sedimentation rate; HRCT, high-resolution computed tomography; IQR, interquartile range; IL-6, interleukin-6; ILD, interstitial lung disease; JAK inhibitors, Janus kinase inhibitors; KL-6, Krebs von den Lungen-6 antigen; LDH, lactate dehydrogenase; mMRC, modified Medical Research Council dyspnoea scale; SP-D, surfactant-associated protein D; SSc, systemic sclerosis; TNF inhibitors, tumour necrosis factor inhibitors; WBC, white blood cell

### Patients’ characteristics stratified according to the radiological severity of ILD

**Fig. 1:**
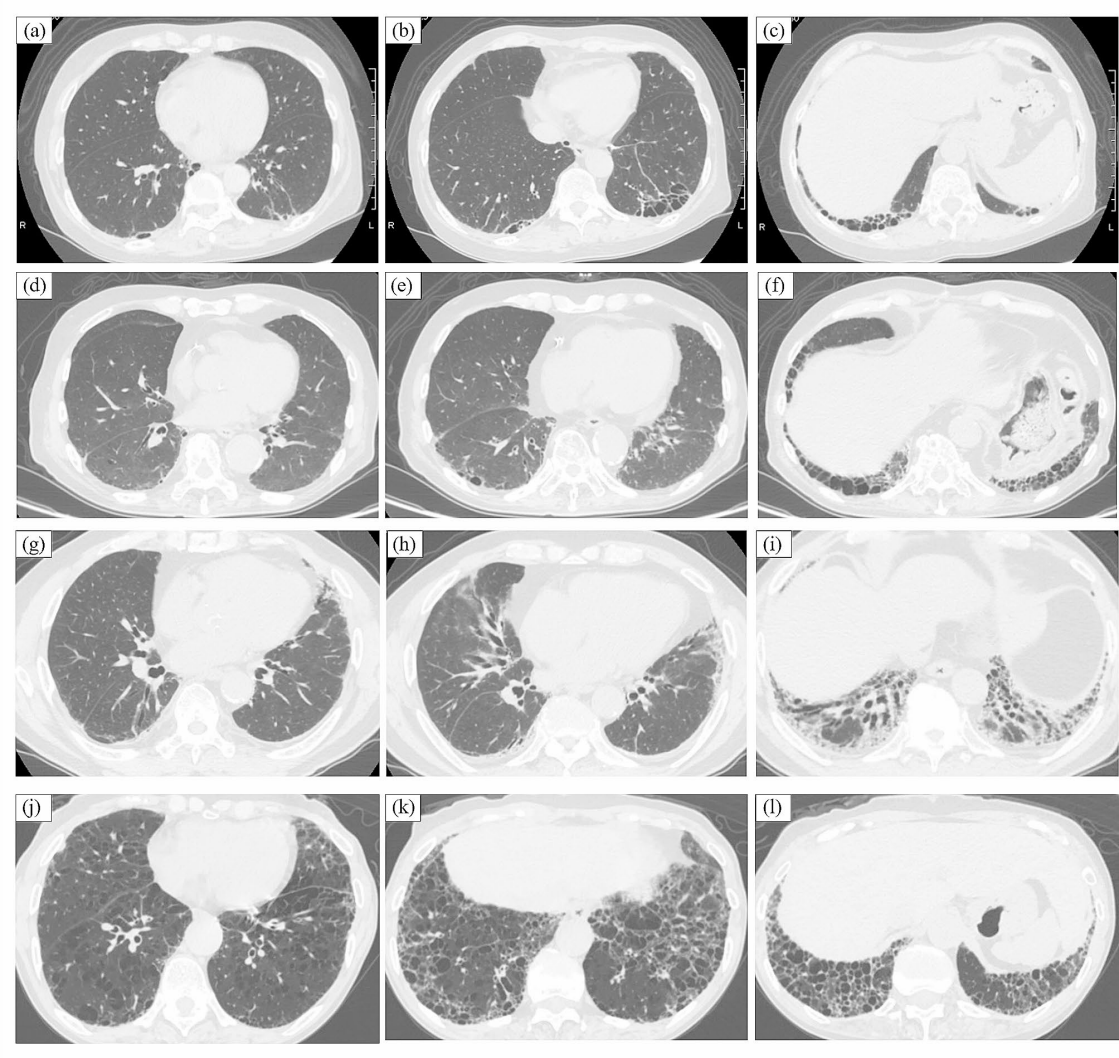
Representative results of quantitative extent of ILD based on the conventional visual scoring method. Case 1: a 62-year-old woman, ILD extent = 10.0% (**a**, **b**, **c**); Case 2: an 83-year-old man, ILD extent = 14.2% (**d**, **e**, **f**); Case 3: a 70-year-old man, ILD extent = 14.2% (**g**, **h**, **i**); Case 4: a 70-year-old man, ILD extent = 27.0% (**j**, **k**, **l**). Slices at the midpoint between the right pulmonary venous confluence and 1 cm above the right hemi-diaphragm dome (**a**, **d**, **g**, **j**), at the 1 cm above the right hemi-diaphragm dome (**b**, **e**, **h**, **k**) and at the 2 cm below the right hemi-diaphragm dome (**c**, **f**, **i**, **l**). Abbreviations; ILD, interstitial lung disease

Patients’ characteristics stratified according to the radiological severity of ILD are listed in Table 2. Among 124 patients, 107 (86.2%) had no ILD, nine (7.2%) had ILD with extent < 10%, three (2.4%) had ILD with extent ≥10% and < 20% and five (4.0%) had ILD with extent ≥20%. Among 17 patients (13.7%) diagnosed with ILD based on HRCT assessment, seven (41.1%) were classified as having usual interstitial pneumonia pattern, 9 (52.9%) as having non-specific interstitial pneumonia pattern. No patient was diagnosed with acute exacerbation of RA-ILD at registration. All patients had chronic ILD at registration, and no patient showed subacute change on ILD which suggest organizing pneumonia (OP) or OP superimposed to preexisting ILD. None of the patients with ILD showed RA-associated bronchial disease (bronchitis or bronchiectasis). Inter-reader agreement for ILD extent was ‘nearly perfect’ with κ = 0.896 (*P* < 0.01) among the 17 patients with ILD. Representative results of the radiological severity of ILD are presented in Fig. 1. Post-stratification, the median values of KL-6 were elevated from 216.0 IU/mL, 345.0 IU/mL, 330.0 U/mL to 949.0 IU/mL in accordance with ILD extent = 0%, ILD extent < 10%, ILD extent ≥10% and < 20% and ILD extent ≥20%, respectively, and the median values of FVC% predicted were decreased from 102.5%, 100.8%, 70.6 to 80.6% in accordance with ILD extent = 0%, ILD extent < 10%, ILD extent ≥10% and < 20% and ILD extent ≥20%, respectively. The median values of DAS28-ESR were elevated from 2.30, 2,45, 3.19 to 4.47 in accordance with ILD extent = 0%, ILD extent < 10%, ILD extent ≥10% and < 20% and ILD extent ≥20%, respectively.

**Table 2:** Patient characteristics stratified according to the radiological severity of interstitial lung disease

	No ILD (ILD extent = 0%) (*n* = 107)	0%< ILD < 10% (*n* = 9)	10%≤ ILD < 20% (*n* = 3)	ILD ≥20% (*n* = 5)
Age, median (IQR)	69.0 [58.5, 74.0]	71.0 [70.0, 75.0]	70.0 [66.0, 76.5]	73.0 [73.0, 78.0]
Sex, n (%)	27 (25.2%)	2 (22.2%)	2 (66.6%)	1 (20.0%)
Disease duration (years), median (IQR)	2.82 [1.35, 8.38]	3.65 [2.18, 5.86]	9.89 [5.52, 20.86]	3.29 [2.62, 5.71]
Ever smoker, n (%)	39 (36.4%)	2 (22.2%)	2 (66.6%)	3 (60.0%)
mMRC ≥1	24 (22.4%)	4 (44.4%)	2 (66.6%)	4 (80.0%)
Cough	14 (13.0%)	5 (55.5%)	2 (66.6%)	4 (80.0%)
KL-6 (U/mL), median (IQR)	216.0 [173.5, 294.5]	345.0 [223.0, 499.0]	330.0 [296.0, 544.0]	949.0 [623.0, 979.0]
SP-D (ng/mL), median (IQR)	62.9 [42.9, 87.0]	82.3 [52.6, 110.1]	85.2 [80.7, 146.4]	93.6 [91.3, 208.6]
Rheumatoid factor, titre (U/mL)	24.0 [5.0, 86.0]	47.0 [18.0, 127.0]	318.0 [262.5, 409.0]	189.0 [157, 500.0]
Anti-CCP antibody, titre (U/mL)	2.4 [1.7, 102.2]	19.1 [1.7, 618.8]	89.4 [83.7, 169.2]	865.0 [387.8, 867.5]
DAS28-ESR, median (IQR)	2.30 [1.54, 3.30]	2.45 [1.81, 3.03]	3.19 [2.40, 4.79]	4.47 [3.68, 5.17]
Pulmonary function tests, available, n	*n* = 66	*n* = 9	*n* = 3	*n* = 5
Forced vital capacity (L), median (IQR)	2.70 [2.24, 3.24]	2.76 [2.30, 2.92]	2.40 [2.28, 2.55]	1.75 [1.66, 2.32]
Forced vital capacity percent predicted (%), median (IQR)	102.5 [94.8, 114.2]	100.8 [95.4, 111.9]	70.6 [68.3, 89.7]	80.6 [71.0, 96.3]
ILD extent (%)	0.00 [0.00, 0.00]	5.00 [3.33, 7.50]	14.17 [12.09, 16.07]	30.42 [29.59, 43.75]
Treatment at registration	MTX 63.5%, bDMARDs 36.4%, JAKi 10.2%	MTX 66.6%, bDMARDs 11.1%, JAKi 44.4%	MTX 66.6%, bDMARDs 33.3%	MTX 40.0%, bDMARDs 40.0%

Abbreviations: bDMARDs, biological disease-modifying anti-rheumatic drugs; DAS28-ESR, disease activity score 28-erhythrocyte sedimentation rate; IQR, interquartile range; JAK inhibitors, Janus kinase inhibitors; ILD, interstitial lung disease; KL-6, Krebs von den Lungen-6 antigen; mMRC, modified Medical Research Council dyspnoea scale; MTX, methotrexate; SP-D, surfactant-associated protein D

### Factors associated with the quantitative extent of ILD

Table 3 presents the univariate and multivariate linear regression analysis of the factors associated with the quantitative severity of ILD. DAS28-ESR (standardized coefficient = 0.295, *P* < 0.01), RF titre (standardized coefficient = 0.318, *P* < 0.01) and anti-CCP antibody titre (standardized coefficient = 0.184, *P* = 0.040) were significantly associated with quantitative extent of ILD in univariate analysis. Among these covariates, RF titre (standardized coefficient = 0.247, *P* = 0.01) and DAS28-ESR (standardized coefficient = 0.199, *P* = 0.03) were significantly associated with the quantitative extent of ILD in multivariate analysis adjusted for age, male sex, smoking status and anti-CCP antibody titre. Supplementary Table S2 shows the results of an exploratory analysis using CDAI as the disease activity index. In univariate analysis, CDAI was significantly associated with quantitative extent of ILD (standardized coefficient = 0.189, *P* = 0.03), although this association did not remain to be significant in multivariate analysis.

**Table 3:** Factors associated with the quantitative extent of ILD

	Univariate analysis		Multivariate analysis	
	Standardized coefficient (95% CI)	*P* value	Standardized coefficient (95% CI)	*P* value
Age	0.144 (-0.032, 0.319)	0.11	0.073 (-0.096, 0.243)	0.39
Male sex	-0.001 (-0.178, 0.177)	0.99	-0.177 (-0.417, 0.063)	0.15
Disease duration (years)	-0.013 (-0.191, 0.164)	0.88	-0.082 (-0.259, 0.096)	0.36
Smoking	0.092 (-0.084, 0.269)	0.30	0.262 (0.015, 0.509)	0.03^a^
DAS28-ESR	0.295 (0.126, 0.465)	< 0.01^a^	0.199 (0.016, 0.382)	0.03^a^
Rheumatoid factor, titre	0.318 (0.150, 0.486)	< 0.01^a^	0.247 (0.060, 0.435)	0.01^a^
Anti-CCP antibody, titre	0.184 (0.010, 0.358)	0.040^a^	0.125 (-0.041, 0.290)	0.14

^a^*P* < 0.05

Abbreviations; anti-CCP antibody, anti-cyclic citrullinated peptide antibody; CI, confidence interval; DAS28-ESR, disease activity score 28-erhythrocyte sedimentation rate; ILD, interstitial lung disease

### Factors associated with FVC% predicted

Univariate and multivariate linear regression analysis of the factors associated with the value of FVC% predicted are presented in Table 4. DAS28-ESR (standardized coefficient = -0.342, *P* < 0.01) and RF titre (standardized coefficient = -0.326, *P* < 0.01) were significantly associated with FVC% predicted in univariate analysis. DAS28-ESR (standardized coefficient = -0.230, *P* = 0.047) was significantly associated with FVC% predicted in multivariate analysis adjusted for age, male sex, smoking status, RF titre and anti-CCP antibody titre. Supplementary Table S3 shows the results of an exploratory analysis using CDAI as the disease activity index. In univariate analysis, CDAI was significantly associated with FVC% predicted (standardized coefficient = -0.195, *P* = 0.049), although this association did not remain to be significant in multivariate analysis.

**Table 4:** Factors associated with forced vital capacity% predicted

	Univariate analysis		Multivariate analysis	
	Standardized coefficient (95% CI)	*P* value	Standardized coefficient (95% CI)	*P* value
Age	-0.202 (-0.418, 0.015)	0.07	-0.121 (-0.329, 0.087)	0.25
Male sex	-0.175 (-0.403, 0.052)	0.13	-0.088 (-0.375, 0.199)	0.54
Disease duration (years)	0.094 (-0.116, 0.304)	0.38	0.155 (-0.051, 0.361)	0.14
Smoking	-0.134 (-0.349, 0.081)	0.22	-0.112 (-0.389, 0.164)	0.42
DAS28-ESR	-0.342 (-0.530, -0.154)	< 0.01^a^	-0.230 (-0.453, -0.006)	0.047^a^
Rheumatoid factor, titre	-0.326 (-0.528, -0.135)	< 0.01^a^	-0.229 (-0.459, 0.002)	0.055
Anti-CCP antibody, titre	-0.067 (-0.248, 0.114)	0.47	-0.010 (-0.176, 0.157)	0.90

^a^*P* < 0.05

Abbreviations; anti-CCP antibody, anti-cyclic citrullinated peptide antibody; CI, confidence interval; DAS28-ESR, disease activity score 28-erhytrocyte sedimentation rate

## Discussion

This study clearly demonstrated that DAS28-ESR was significantly associated with the quantitative severity of RA-ILD for the first time. Our study design is novel in that we enrolled consecutive patients with RA irrespective of the presence/absence of ILD and the severity of ILD was further quantified radiologically from 0 to 100% and investigated the relationship between RA disease activity and severity of comorbid ILD. Our study is more detailed than previous reports in the following three respects regarding the radiological evaluation. First, we adopted not 3-slices [13] evaluation but 6-slice evaluation [14]. Second, we investigated not only GGO [13] but also reticulation and honeycombing [5, 6, 15]. Third, as a semi-quantitative evaluation, lesion extent in each slice was evaluated with 21 grades in 5% increments [5, 14] instead of 6 grades [13].

In our study, consecutive patients with RA were analysed irrespective of the presence/absence of ILD and numerically scored for the severity of ILD radiologically from 0 (no involvement) to 100% (all lung fields affected). Previous reports on the relationship between RA disease activity and ILD are mainly analysed by two ways: (1) investigate the relationship between disease activity and the presence/absence of comorbid ILD, (2) investigate the relationship between disease activity and severity of ILD among patients having RA-ILD. Among the reports using the former way, Restrepo et al. showed that for each one increase in DAS28-ESR, the odds ratio of having ILD increased by 1.49 [11], and Chen et al. compared cases with and without ILD and demonstrated that DAS28-ESR was significantly higher in the ILD group [10]. Regarding reports using the latter way, the aforementioned report by Pérez-Dórame et al. is available, but there are some limitations in terms of radiological evaluation [13]. In real clinical practice, complications of ILD range from very mild ILD, which is clinically insignificant, to severe ILD, which has a major impact on treatment strategies and prognosis. In our study, we also included patients without ILD in our analysis, converting the presence and severity of ILD into continuous variables ranging from 0% (no involvement) to 100% (all lung fields affected). By adopting this unique research design, we could demonstrate the relationship between disease activity and severity of comorbid ILD in more detail than previously reported [10, 11, 13]. Furthermore, we also demonstrated a significant correlation between RA disease activity and ILD severity on physiological parameter. No previous study has investigated on the relationship between RA disease activity and physiological severity of ILD.

To date, one report by Pérez-Dórame et al. has investigated the relationship between RA disease activity and radiological severity of RA-ILD [13]. In this previous study, slices number to be evaluated were three, investigated lung lesion was limited to GGO, and assessment of each slice was based on 6 grade-semi quantification [13]. On the contrary, in our study, slices number to be evaluated were six, investigated lung lesion was combined extent of GGO, reticulation and honeycombing, and assessment of each slice was based on 21 grade-semi quantification with 5% increments. The strength of this study is that we performed a detailed image evaluation compared to the previous report [13]. Furthermore, as our study uses a currently established method [14, 15] to assess ILD severity, the results obtained here regarding the relationship between comorbid ILD severity and disease activity are of high clinical value. Previous reports have shown that the severity of RA-ILD, as assessed by the currently established method used in this study, has a significant correlation with its prognosis [4–6].

The number of patients with ILD with extent > 20% was relatively low compared with that in previous reports [5, 6]. One reason for the low incidence of severe ILD may be that the disease duration was relatively short in this cohort compared to those in previous studies [10, 11]. Since this study enrolled consecutive patients who visited our rheumatology centre, it is possible that more patients with RA at an earlier stage were present compared to the general cohort. Conversely, a significant correlation between disease activity and severity of comorbid ILD observed in this study may attribute to the present characteristics of this study population that many patients had untreated comorbid ILD. In our study, known risk factors of RA-ILD [25], such as age, disease duration, and smoking status, were not significantly associated with the quantitative extent of ILD. This might partly be attributable to the active treatment for RA that participants received prior to study enrolment.

This study has some limitations. First, this was a single-centre study with a relatively small sample size. Patients with ILD included in this study were relatively small. Second, while all patients with ILD underwent pulmonary function tests, not all patients without ILD underwent pulmonary function tests. Third, the present results may be influenced by the relatively large proportion of patients with late-onset RA who were negative for anti-CCP antibodies and were more likely to be treated with prednisolone. These features were reported to be the characteristic findings in patients with late-onset RA [26]. Forth, since this study was a cross-sectional study, it was not possible to examine how changes in disease activity due to treatment affect the course of ILD. Further studies are needed to overcome these limitations in this study.

## Conclusions

Our study revealed for the first time that RA disease activity significantly impacts on the severity of ILD both on radiologically and physiologically.

### Electronic supplementary material

Below is the link to the electronic supplementary material.

Supplementary Material 1

## Data Availability

Data may be made available upon valid request to the corresponding author.

## Abbreviations

ANA: antinuclear antibody
anti-CCP: antibody anti-cyclic citrullinated peptide antibody
CI: confidence interval
CTD: connective tissue disease
DAS: disease activity score
ESR: erythrocyte sedimentation rate
FVC: forced vital capacity
GGO: ground glass opacity
HRCT: high-resolution computed tomography
ILD: interstitial lung disease
IQR: interquartile range
JAK: inhibitors Janus kinase inhibitors
KL-6: Krebs von den Lungen-6 antigen
OP: organizing pneumonia
RA: rheumatoid arthritis
RF: rheumatoid factor
