# Supplementary material for: Rheumatoid arthritis disease activity significantly impacts on the severity of interstitial lung disease
Source: Arthritis Res Ther. 2024 May 4;26:95. doi: 10.1186/s13075-024-03333-6 (PMC11069302; doi:10.1186/s13075-024-03333-6)
Supplement: Supplementary file 1 — Supplementary Material 1 [file 13075_2024_3333_MOESM1_ESM.docx]

**Supplementary Data S1**
**Conventional visual scoring method for the radiological severity of interstitial lung disease (ILD)**

*High-resolution computed tomography (HRCT) protocol and image evaluation*

Upon registration at our institution, all participants underwent chest HRCT, using multidetector-row CT (MDCT; Revolution CT; GE Healthcare, Milwaukee, WI; LightSpeed VCT (GE Healthcare), SOMATOM Force (Siemens Healthcare, Erlangen, Germany), or SOMATOM Definition Flash (Siemens Healthcare)). The lung apex–base HRCT images were acquired at suspended full inspiration. The CT scan parameters included a tube voltage of 120 kVp and auto-exposure control. The CT images were reconstructed with a slice thickness of 1.00 or 1.25 mm, depending on the CT system. For the assessment of lung parenchyma, the HRCT images were displayed with a default window setting (window width and level: 1700 and -700 Hounsfield units, respectively). A radiological diagnosis for each case was determined by selecting from usual interstitial pneumonia, non-specific interstitial pneumonia, and other CT patterns, according to Walsh et al.’s method [1]. This radiological diagnosis was based on the current international consensus statement of idiopathic interstitial pneumonias [2].

*Extent of ILD*

The ILD score was calculated as follows. First, six levels in each HRCT scan were preselected according to Walsh et al.’s method [1]. Walsh et al. classified interstitial abnormalities into three categories: ground glass opacities, reticulation, and honeycombing, and calculated the scores for each [1]. In this study, we calculated the score as the sum of these three types of interstitial abnormalities according to Goh et al.’s method [3]. Definition of interstitial abnormalities were based on the definition by Walsh et al. [1, 4]. Second, the extent of ILD was calculated as the ratio of the combined area of these three interstitial abnormalities to the total lung field scored visually in 5% increments on the six HRCT slices, with the total score divided by six.

*Definition of interstitial abnormalities*

1) Ground glass opacity: increased parenchymal density with preservation of the bronchial and vascular markings, with or without superimposed extremely fine texture albeit without obvious reticulation.

2) reticulation: criss-crossing linear opacities that are fine or coarse (including inter- or intra-lobular septal thickening) with associated distortion of the lung architecture.

3) honeycombing: air-filled cystic spaces with irregular walls deemed not to represent traction bronchiectasis.

**Supplementary Table S1. Classification criteria for ILD in RA**

| Probable ILD | HRCT report containing terms such as ‘pulmonary fibrosis’, ‘fibrotic changes’, ‘fibrosis’, ‘RA-lung’, ‘fibrosing alveolitis’, and presence of nonspecific abnormalities that can be observed in ILD  and  Treating physician’s diagnosis of ‘pulmonary fibrosis’, ‘RA-lung’, ‘fibrosing alveolitis’, or other terms in the medical records consistent with ILD |
| --- | --- |
| Definite ILD | Diagnosis of ILD by a pulmonologist  and  Two of the following three criteria:  ILD observed on HRCT  Restrictive pattern observed on PFT (TLC ≤80% predicted)  Bronchoscopy or surgical lung biopsy results consistent with ILD |

Abbreviations: ILD, interstitial lung disease; HRCT, high-resolution computed tomography; PFT, pulmonary function testing; RA, rheumatoid arthritis; TLC, total lung capacity. In reference to the report by Bongartz et al. with minor modification [5], ILD is classified as probable ILD or definite ILD in the present study.

**Supplementary Table S2. Factors associated with the quantitative extent of ILD**

|  | Univariate analysis |  | Multivariate analysis |  |
| --- | --- | --- | --- | --- |
|  | Standardized coefficient (95% CI) | *P* value | Standardized coefficient (95% CI) | *P* value |
| Age | 0.144 (-0.032, 0.319) | 0.11 | 0.091 (-0.081, 0.263) | 0.30 |
| Male sex | -0.001 (-0.178, 0.177) | 0.99 | -0.157 (-0.400, 0.086) | 0.20 |
| Disease duration (years) | -0.013 (-0.191, 0.164) | 0.88 | -0.080 (-0.261, 0.101) | 0.38 |
| Smoking | 0.092 (-0.084, 0.269) | 0.30 | 0.232 (-0.018, 0.481) | 0.07 |
| CDAI | 0.189 (0.016, 0.363) | 0.03^a^ | 0.080 (-0.096, 0.256) | 0.37 |
| Rheumatoid factor, titre | 0.318 (0.150, 0.486) | <0.01^a^ | 0.298 (0.111, 0.484) | <0.05^a^ |
| Anti-CCP antibody, titre | 0.184 (0.010, 0.358) | 0.040^a^ | 0.138 (-0.031, 0.306) | 0.11^a^ |

^a^ *P* < 0.05
Abbreviations; anti-CCP antibody, anti-cyclic citrullinated peptide antibody; CDAI, clinical disease activity index; CI, confidence interval.

**Supplementary Table S3. Factors associated with forced vital capacity% predicted**

|  | Univariate analysis |  | Multivariate analysis |  |
| --- | --- | --- | --- | --- |
|  | Standardized coefficient (95% CI) | *P* value | Standardized coefficient (95% CI) | *P* value |
| Age | -0.202 (-0.418, 0.015) | 0.07 | -0.149 (-0.361, 0.064) | 0.17 |
| Male sex | -0.175 (-0.403, 0.052) | 0.13 | -0.112 (-0.406, 0.181) | 0.45 |
| Disease duration (years) | 0.094 (-0.116, 0.304) | 0.38 | 0.168 (-0.043, 0.379) | 0.12 |
| Smoking | -0.134 (-0.349, 0.081) | 0.22 | -0.078 (-0.361, 0.205) | 0.58 |
| CDAI | -0.195 (-0.386, -0.003) | 0.049^a^ | -0.072 (-0.278, 0.135) | 0.49 |
| Rheumatoid factor, titre | -0.326 (-0.528, -0.135) | <0.01^a^ | -0.317 (-0.542, -0.092) | <0.01^a^ |
| Anti-CCP antibody, titre | -0.067 (-0.248, 0.114) | 0.47 | -0.021 (-0.193, 0.150) | 0.80 |

^a^ *P* < 0.05
Abbreviations; anti-CCP antibody, anti-cyclic citrullinated peptide antibody; CDAI, clinical disease activity index; CI, confidence interval.

**References**

1. Walsh SL, Sverzellati N, Devaraj A, Keir GJ, Wells AU, Hansell DM: **Connective tissue disease related fibrotic lung disease: high resolution computed tomographic and pulmonary function indices as prognostic determinants**. *Thorax* 2014, **69**(3):216-222.

2. Travis WD, Costabel U, Hansell DM, King TE, Jr., Lynch DA, Nicholson AG, Ryerson CJ, Ryu JH, Selman M, Wells AU *et al*: **An official American Thoracic Society/European Respiratory Society statement: Update of the international multidisciplinary classification of the idiopathic interstitial pneumonias**. *Am J Respir Crit Care Med* 2013, **188**(6):733-748.

3. Goh NS, Desai SR, Veeraraghavan S, Hansell DM, Copley SJ, Maher TM, Corte TJ, Sander CR, Ratoff J, Devaraj A *et al*: **Interstitial lung disease in systemic sclerosis: a simple staging system**. *Am J Respir Crit Care Med* 2008, **177**(11):1248-1254.

4. Hansell DM, Bankier AA, MacMahon H, McLoud TC, Muller NL, Remy J: **Fleischner Society: glossary of terms for thoracic imaging**. *Radiology* 2008, **246**(3):697-722.

5. Bongartz T, Nannini C, Medina-Velasquez YF, Achenbach SJ, Crowson CS, Ryu JH, Vassallo R, Gabriel SE, Matteson EL: **Incidence and mortality of interstitial lung disease in rheumatoid arthritis: a population-based study**. *Arthritis Rheum* 2010, **62**(6):1583-1591.
